# Depressive symptoms in hospitalized geriatric patients with and without cognitive impairment: a cross-sectional network analysis approach

**DOI:** 10.3389/fmed.2026.1689283

**Published:** 2026-03-16

**Authors:** Tino Prell, Aline Schönenberg, Konstantin G. Heimrich

**Affiliations:** 1 Department of Geriatrics, Halle University Hospital, Halle (Saale), Germany; 2 Department of Geriatric Medicine, Jena University Hospital, Jena, Germany

**Keywords:** cognitive impairment, depression, geriatric assessments, oldest-old, symptom network

## Abstract

**Background:**

Late-life depression is common and often co-occurs with cognitive impairment, complicating its assessment and clinical management. Network analysis allows for a nuanced understanding of how individual depressive symptoms interact. This study examines differences in the network structure of depressive symptoms in geriatric patients with and without cognitive impairment.

**Methods:**

We included monocentric cross-sectional data of 3,990 hospitalized geriatric inpatients whose depressive symptoms were rated using the 15-item Geriatric Depression Scale (GDS). Patients were stratified into an unimpaired and impaired cognition group depending on the Mini-Mental State Examination (MMSE) (cut-off < 24 points). Network analyses were estimated separately for both groups using regularized logistic regression models. A network comparison test was conducted for group comparison.

**Results:**

Our study showed that worthlessness was the most central depressive symptom. However, the network structures differed notably between the two groups, with less impact of feeling empty when cognitive impairment is present, as well as a stronger association between feeling unsatisfied and unhappy, and a weaker connection between feeling empty and bored.

**Conclusion:**

These differences highlight the need for clinicians and public health professionals to adapt their screening and intervention strategies to take into account the subtle presentation of depressive symptoms in older adults according to cognitive status.

## 1 Introduction

Late-life depression is a prevalent and debilitating condition, affecting up to one third of older adults and frequently complicating the course of neurocognitive disorders (1). In routine geriatric care, depressive symptoms are commonly assessed using summative self-report instruments such as the 15-item Geriatric Depression Scale (GDS-15), which captures affective, cognitive, and somatic aspects of depression (2). While widely used, traditional latent variable approaches conceptualize depression as an underlying construct reflected by observed symptoms, thereby offering limited insight into the direct interrelations among individual depressive features. Over the past decade, network analysis has been increasingly applied in psychopathology research as a complementary framework that conceptualizes mental disorders as systems of interacting symptoms rather than as expressions of a single latent cause (3, 4). In geriatric depression research, network approaches have been used to explore symptom structure and to identify potentially influential symptoms within depressive presentations. Studies employing the GDS-15 in cognitively intact older adults have shown that affective symptoms such as sadness, hopelessness, or feelings of worthlessness often occupy central positions within symptom networks and are strongly connected to other depressive features (5).

However, depressive symptomatology in the context of cognitive impairment may differ substantially from that observed in cognitively unimpaired individuals. Neurocognitive deficits can affect emotional awareness, self-report accuracy, and symptom expression, potentially leading to reduced endorsement of affective distress and relative prominence of apathetic, motivational or somatic symptoms (6, 7). Such differences are likely to influence not only the prevalence of individual symptoms but also their pattern of interrelations within depressive symptom networks (8). Despite growing interest in network-based approaches, comparative item-level analyses of depressive symptom networks stratified by cognitive status remain limited. In particular, it is unclear whether and how the structure, connectivity, and centrality of GDS-15 symptoms differ between geriatric patients with and without cognitive impairment. Addressing this gap is clinically relevant given the complex and bidirectional relationship between depression and cognitive impairment, which continues to pose challenges for diagnosis, treatment, and the optimization of functional outcomes and the quality of life in older adults (9, 10).

In the present study, we conducted a large-scale, item-level network analysis of depressive symptoms in hospitalized geriatric patients with and without cognitive impairment, using the same assessment instrument (GDS-15) across groups. We estimated and formally compared symptom networks to examine differences in global connectivity, edge-level associations, and symptom centrality as a function of cognitive status. By adopting a comparative and hypothesis-refining approach grounded in routine geriatric care data, this study aims to advance understanding of how cognitive impairment shapes the organization of depressive symptoms, thereby informing more differentiated assessment and potentially more tailored clinical interventions in late-life depression.

## 2 Materials and methods

### 2.1 Study design

We retrospectively analyzed monocentric routine data from the Department of Geriatrics, University Hospital Jena, Germany. We included patients who received treatment within comprehensive geriatric care (CGC) between January 2014 and December 2023. Patients with severe cognitive impairment [Mini-Mental State Examination (MMSE) score of 16 points or less, or an indeterminate score (11), for example due to obvious delirium] were excluded because the validity of self-reported depression screening instruments is limited in this population (12).

The study was conducted in line with the Declaration of Helsinki and received approval from the local ethics committee of the University Hospital Jena (registration number: 2024-3252-Daten). Since it was a retrospective study using anonymized data, the ethics committee waived the need for patient consent. The authors did not have access to any information that could potentially identify individual study patients.

### 2.2 Study population

**FIGURE 1:**
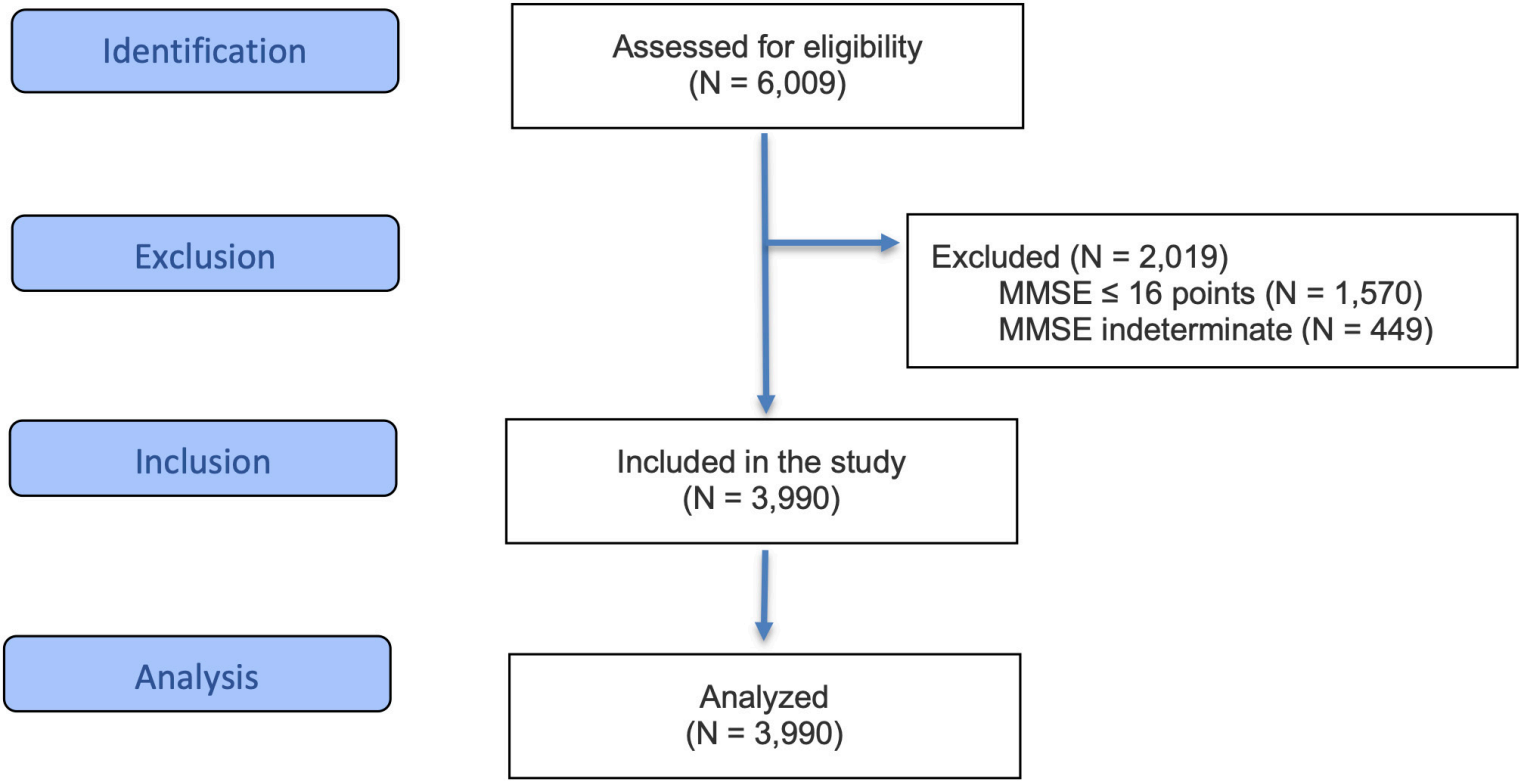
Flow diagram of the study cohort. MMSE, Mini-Mental State Examination.

During the study period, a total of *N* = 6,009 hospitalized patients were treated within the CGC. The majority of these cases were related to cardiopulmonary, urogenital, musculoskeletal, and neurological diseases. Of those, *N* = 2,019 (33.6%) were excluded due to severe cognitive impairment. Of the excluded patients, *N* = 1,570 (26.1%) scored 16 points or less on the MMSE, and *N* = 449 (7.5%) were not determinable. Accordingly, *N* = 3,990 geriatric patients were included in the analyses (see Figure 1). Despite different primary diagnoses, patients were assessed for physical and psychological limitations and capabilities to enable better comparability.

### 2.3 Dependent variables

The GDS-15 is one of the most popular scales for assessing late-life depression and most frequently used within CGC in Germany (2, 13, 14). Its corresponding 15 items captures a range of affective, cognitive, and somatic aspects of depression (2, 13). Each item was read to the patient and they were asked to answer with “yes” or “no.” A response of “yes” indicated the presence of depression in 10 of the 15 items, whereas the remaining five items were reverse coded (Items 1, 5, 7, 11, and 13).

### 2.4 Independent variables and covariates

Cognitive status was determined by the MMSE, dichotomized at a cut-off of 24 points to form a “unimpaired cognition” (UC) group (MMSE ≥ 24; *N* = 2,625) and a “impaired cognition” (IC) group with mild to moderate cognitive impairment (MMSE 17–23; *N* = 1,365) (11).

To describe the cohort, age (metric), sex (male, female), and self-help capability according to the Barthel Index [ranging from 0 (totally dependent) to 100 (totally independent)] (15) were also recorded. This data was collected during the inpatient stay and compiled retrospectively for this study.

### 2.5 Statistical analyses

All statistical analyses were performed in R version 4.1.1. Descriptive statistic was used to characterize the cohort. For group comparisons, independent *t*-tests and chi-square tests were performed. The effect sizes were given as Cohen’s d for the independent *t*-test and Cramer’s V for the chi-square test. The effect sizes were considered as low (| 0.2|), moderate (| 0.5|), or high (| 0.8|) (16).

We estimated a network for the entire cohort and two separate symptom networks (one per cognitive group). Missing data of GDS items were deleted listwise. We estimated the network with the *IsingFit* package (17), using an ℓ1-regularized logistic regression with tuning parameter γ = 0.5 to impose sparsity and avoid overfitting. The resulting weighted adjacency matrices quantify the unique pairwise conditional associations between symptoms, controlling for all others. Networks were visualized in the *qgraph* package (18). To identify the most influential symptoms within each weighted, undirected network, we computed node strength (the sum of absolute edge weights attached to a node) via the *bootnet* package (19). Nodes with a high strength centrality are directly connected to other symptoms. These highlighted symptoms could therefore have far-reaching downstream effects if they are addressed therapeutically. Network stability was estimated with a case-dropping bootstrap procedures (1,000 resamples) in *bootnet* (19). The correlation stability (CS) coefficients were computed to ensure that the estimates of strength remained stable among different participant groups (CS ≥ 0.25) (19).

Furthermore, we aimed to evaluate the differences between the networks of people with unimpaired (UC) and impaired cognition (IC). Therefore, we performed a network comparison test with 1,000 permutations to assess network structure invariance (the maximum difference in pairwise edges) and global strength invariance (the difference in the weighted absolute sum of all edges) between the UC and IC networks using the *NetworkComparisonTest* package (20). In addition to omnibus tests, we examined specific edge-weight and centrality differences, correcting for multiple comparisons via permutation-based *p*-values.

To assess the robustness of the network estimates, several sensitivity analyses were conducted. First, to evaluate the potential impact of missing GDS-15 item data, network estimation was repeated using multiple imputation at the item level with the *mice* package (21). Missing binary GDS items were imputed using logistic regression models with five imputed datasets, and network centrality estimates were averaged across imputations and compared with the complete-case solution. Second, to examine the influence of the regularization parameter on network structure and centrality, Ising models were re-estimated using a range of EBIC tuning parameters (γ = 0.25, 0.50, and 0.75). The stability of node centrality rankings and key edge weights across these specifications was inspected. Third, to assess whether centrality patterns depended on the specific estimation framework, we conducted an additional sensitivity analysis using mixed graphical models (mgm) as an alternative approach for binary data with the *mgm* package (22). The mgm framework estimates conditional dependencies via nodewise regularized regressions and thus differs conceptually from the penalized Ising model implemented in *IsingFit*. Networks were estimated using categorical variables with two levels per item. From the resulting weighted adjacency matrix, node strength was calculated as the sum of absolute edge weights per node. Centrality estimates obtained from the mgm-based network were compared with those derived from the IsingFit model using rank-order correlations. Finally, for the group comparison between UC and IC patients, sensitivity analyses focused on the robustness of group differences in key edges identified by the network comparison test. These edges were re-estimated across different regularization parameters to evaluate the consistency of direction and magnitude of between-group differences.

## 3 Results

The characteristics of the cohort are given in Table 1. People with cognitive deficits were older, had poorer Barthel Index and more depressive symptoms. However, the effect sizes for these differences were weak or modest (Table 2).

**TABLE 1:** Characteristics of the studied cohort (*N* = 3,990).

Variables		Missings (*N*)	Values	
Age (M, SD)		0	82.61	6.51
Sex (*N*, %)	Male	0	1,367	34.3
	Female	0	2,623	65.7
MMSE (M, SD)		0	24.58	3.29
MMSE (*N*, %)	24–30 points	0	2,625	65.8
	23–17 points	0	1,365	34.2
Barthel index (M, SD)		4	50.18	21.10
GDS (M, SD)		101	4.27	2.94
**GDS item present (*N*, %)**				
G_1i: satisfied* (unsatisfied)		101	626	16.1
G_2: dropped interests		101	2,223	57.1
G_3: empty		101	906	23.3
G_4: bored		101	770	19.8
G_5i: good spirits* (bad spirits)		101	731	18.8
G_6: afraid		101	925	23.8
G_7i: happy* (unhappy)		101	375	9.6
G_8: helpless		101	1,465	37.7
G_9: stay home		101	2,594	66.7
G_10: memory problems		101	784	20.2
G_11i: wonderful* (awful)		101	672	17.3
G_12: Worthless		101	759	19.5
G_13i: energy* (lack of energy)		101	2,470	63.5
G_14: hopeless		101	619	15.9
G_15: others better off		101	664	17.1

Metric values are presented as mean (M) and standard deviation (SD); categorical parameters are presented as numbers (*N*) and percentages (%). GDS, Geriatric Depression Scale; MMSE, Mini-Mental State Examination.

*Inversely coded items of the GDS (G_1i, G_5i, G_7i, G_11i, and G_13i).

**TABLE 2:** Group comparison of people with unimpaired (UC) and impaired cognition (IC).

Variables	Missings (*N*)	Unimpaired Cognition (*N* = 2,625)	Impaired cognition (*N* = 1,365)	*P*	*r*
Age (M, SD)	0	82.29 ± 6.54	83.21 ± 6.41	<0.001	−0.14
MMSE (M, SD)	0	26.59 ± 1.68	20.71 ± 1.87	<0.001	3.36
Barthel index (M, SD)	4	54.31 ± 19.72	42.24 ± 21.39	<0.001	0.60
GDS (M, SD)	101	4.13 ± 2.88	4.56 ± 3.04	<0.001	−0.15
Sex (male/female) (*N*, %)	0	878 (64.2)/1,747 (66.6)	489 (35.8)/876 (33.4)	0.133	/

Metric values are presented as mean (M) and standard deviation (SD); categorical parameters are presented as numbers (*N*) and percentages (%). For group comparisons, independent *t*-tests or chi-square tests were performed and effect sizes were given as Cohen’s d or Cramer’s V. GDS, Geriatric Depression Scale; MMSE, Mini-Mental State Examination.

Complete information on all 15 GDS items was available for 3,889 out of the 3,990 people (97.5%). In contrast, data for all GDS items was missing for 101 people (2.5%) because the GDS had not been administered in these cases. As these people provided no item-level information, the missing GDS data were handled via listwise deletion, and all network analyses were conducted on the complete-case sample.

The network structure for the entire cohort (people with unimpaired and impaired cognition) is given in Supplementary Figure 1 and the node strength of the cohort in Supplementary Table 1. In the network estimated for the entire cohort, feelings of “worthlessness” (G_12) and “emptiness” (G_3) stand out as the most central symptoms. “Worthlessness” (G_12) had the strongest connections to “emptiness” (G_3), “hopelessness” (G_14), and “helplessness” (G_8). Conversely, symptoms reflecting social withdrawal (G_9) and perceived memory problems (G_10) exhibiting minimal connectivity to other nodes.

**FIGURE 2:**
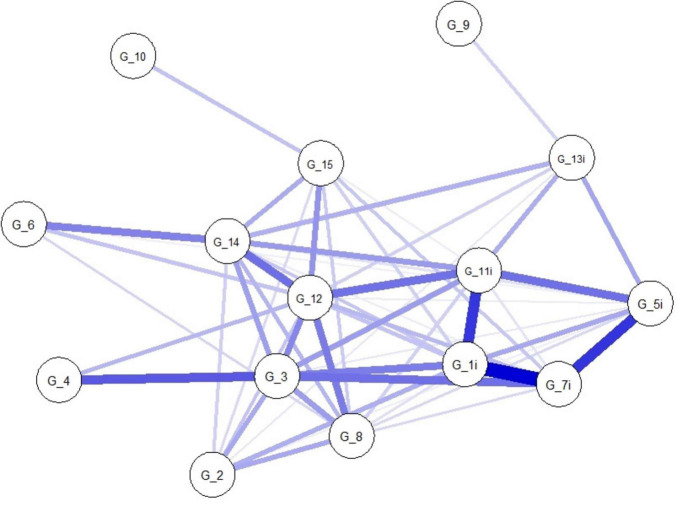
Network plot of people with unimpaired cognition (UC) (*N* = 2,580). Network structure of the GDS-15 items in people with unimpaired cognition. The nodes display the items of the GDS (G_1–G_15). The blue edges display the correlations between the nodes. The thickness of the edges indicate how strong these connections are. GDS, Geriatric Depression Scale. Item coding G_1i: unsatisfied; G_2, dropped interests; G_3, empty; G_4, bored; G_5i, bad spirits; G_6, afraid; G_7i, unhappy; G_8, helpless; G_9, stay home; G_10, memory problems; G_11i, awful; G_12, worthless; G_13i, lack of energy; G_14, hopeless; G_15, others better off.

**FIGURE 3:**
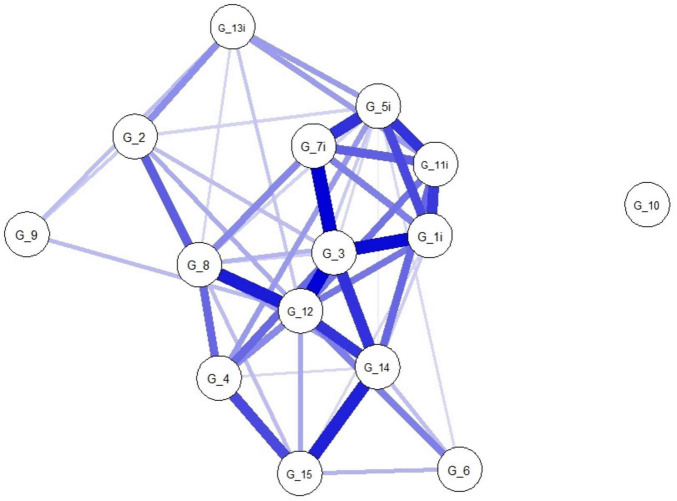
Network plot of people with impaired cognition (IC) (*N* = 1,309). Network structure of the GDS-15 items in people with impaired cognition. The nodes display the items of the GDS (G_1–G_15). The blue edges display the correlations between the nodes. The thickness of the edges indicate how strong these connections are. GDS, Geriatric Depression Scale. Item coding G_1i, unsatisfied; G_2, dropped interests; G_3, empty; G_4, bored; G_5i, bad spirits; G_6, afraid; G_7i, unhappy; G_8, helpless; G_9, stay home; G_10, memory problems; G_11i, awful; G_12, worthless; G_13i, lack of energy; G_14, hopeless; G_15, others better off.

The network plots of people with unimpaired (*N* = 2,580) and impaired cognition (*N* = 1,309) are given in Figures 2, 3, and the corresponding strength centrality plots in Supplementary Figures 2, 3. The Network Comparison Test indicated that the overall structure of the GDS-15 symptom network differs significantly between geriatric patients with unimpaired and impaired cognition (network structure invariance: M = 0.94, *p* = 0.012), whereas the total connectivity (network global strength invariance) does not (S = 3.87, *p* = 0.543), implying that while the pattern of symptom-symptom associations shifts with cognitive status, the average magnitude of those associations remains constant. At the edge level, two symptom-pairs showed significantly different associations after 1,000 permutations: the link between “unsatisfied” (G_1i) and “unhappy” (G_7i) was markedly stronger in the impaired cognition (IC) group (E = 0.94, *p* = 0.004), and the connection between “empty” (G_3) and “bored” (G_4) was also significantly altered (E = 0.50, *p* = 0.033). No other edges survived correction for multiple testing. Finally, centrality invariance tests revealed that only the centrality of “empty” (G_3) decreased significantly when cognitive impairment is present (Strength: *p* = 0.041), indicating that this symptom’s relative importance within the network is reshaped by cognitive decline.

Bootstrap analyses confirmed the stability of these findings. Case-dropping bootstraps yielded consistent estimates of strength centrality exceeding high thresholds in both groups [UC: CS (cor = 0.7) = 0.75; IC: CS (cor = 0.7) = 0.59] as shown in Supplementary Figures 4, 5.

Together, these results suggest that, while cognitive impairment leaves the overall symptom connectivity intact, it alters the pattern of symptom interrelations, particularly by reinforcing the link between life satisfaction and happiness, and by modifying how emptiness and boredom co-occur. The changing centrality of “empty” indicates that its role in the depressive network changes with the onset of cognitive impairment.

Sensitivity analyses addressing missing data yielded results highly consistent with the primary complete-case analyses (Supplementary Table 2), indicating that the observed network structure and centrality patterns were robust to the handling of missing item-level data. Sensitivity analyses varying the EBIC regularization parameter (γ = 0.25, 0.50, and 0.75) yielded highly comparable node strength estimates, with unchanged rank ordering of central symptoms, indicating that the identified network structure was not driven by oversparsification (Supplementary Table 3). Sensitivity analyses using an alternative network estimation based on mgm yielded highly comparable centrality patterns, with an almost perfect rank-order correlation between node strength estimates from the IsingFit and mgm approaches (Spearman’s ρ = 0.996; Supplementary Table 4). Sensitivity analyses were performed to examine the stability of key between-group edge differences across varying regularization parameters (γ = 0.25, 0.50, 0.75) (Supplementary Table 5). Although absolute edge weights varied slightly with the degree of regularization, the direction and relative magnitude of group differences remained stable across all specifications, supporting the robustness of these findings beyond statistical significance.

## 4 Discussion

Our analysis revealed a significant difference in network structure between geriatric patients with and without cognitive impairment, although overall global connectivity (global network strength) was comparable between groups. In both networks, symptoms reflecting diminished self-worth and meaning – most notably feeling “worthless” and feeling that life is “empty” – emerged as highly central nodes. While “worthlessness” retained a central role regardless of cognitive status, “emptiness” appeared to be less influential in the presence of cognitive impairment. Additionally, two symptom-to-symptom associations differed significantly between groups: the connection between “satisfied” and “happy,” and between “empty” and “bored.” These findings suggest that cognitive impairment alters the way specific depressive symptoms co-occur or reinforce one another, even when overall connectivity remains unchanged.

Our findings are consistent with and extend previous network analyses of late-life depression. For example, Kim et al. identified a similar pattern of central depressive symptoms in older adults assessed with the GDS-15 (5). In their study, “worthless,” “empty,” “hopeless”, and “bored” exhibited the highest centrality scores, with the strongest single association observed between “empty” and “bored” (5). This aligns with our observation that the Empty–Bored connection represents a key differentiating feature between cognitively unimpaired and impaired individuals. Together, these results suggest that feeling life is empty and feeling bored are tightly linked in late-life depression, while the strength of this linkage may be modulated by cognitive status. Kim et al. also reported “happy” as a centrally connected symptom and identified “satisfied” as having high discriminative value for depression severity (5). This provides important context for our finding that the Satisfied–Happy edge differed between groups, indicating that cognitive impairment may influence how life satisfaction and happiness relate to one another.

Further support for our findings comes from a study by Hong et al., which specifically examined depression symptom networks in older adults with mild cognitive impairment (MCI) or early dementia (8). In their network, “helplessness” emerged as the most central symptom and served as a major hub with strong connections to symptoms such as restlessness and fatigue (8). Although “helplessness” did not constitute the most central node in our analysis, feelings of worthlessness (conceptually related to helplessness through shared elements of diminished self-esteem) were highly central in both cognitive groups. Moreover, the symptom pattern observed in our cognitively impaired group is broadly consistent with the findings of Hong et al., suggesting a greater emphasis on immediate distress (e.g., helplessness and worthlessness) and a reduced prominence of more abstract emotions such as emptiness (8).

Taken together, these converging findings indicate that affective-cognitive symptoms reflecting feelings of hopelessness, helplessness or low self-esteem act as central hubs in late-life depressive symptom networks. However, the relative prominence of specific symptoms appears to shift depending on cognitive status. Hopelessness, defined as a negative outlook on the future, and helplessness, characterized by a sense of powerlessness in the present, are closely related yet distinct constructs. Cognitive impairment may bias the depressive experience away from future-oriented hopelessness toward more present-focused helplessness, as suggested by Hong et al. (8). In line with this interpretation, our finding that “emptiness” (a symptom conceptually related to hopelessness) lost centrality in the cognitively impaired group, while “worthlessness” remained central, supports this interpretation.

One plausible explanation for these network differences lies in altered symptom perception and reporting associated with cognitive decline. Cognitive decline is often accompanied by reduced insight into one’s emotional state and by affective blunting, characterized by diminished emotional intensity or expression (7). Older adults in general are less likely than younger individuals to endorse cognitive-affective depression symptoms such as pronounced sadness or guilt (6, 23). In addition, apathy, defined by reduced initiative, interest, and emotional expression, is highly prevalent in both late-life depression and cognitive disorders (24). Such apathy may attenuate the subjective experience of sadness or emptiness, even in the presence of observable withdrawal or disengagement. Consequently, cognitively impaired individuals may be less likely to verbalize existential feelings such as emptiness or hopelessness due to limited insight or emotional awareness (25). Instead, depressive symptoms may manifest more prominently as inactivity, irritability, or a general sense of helplessness related to difficulties in performing everyday tasks. This pattern corresponds closely to Hong et al.’s identification of helplessness as a central symptom in cognitively impaired older adults (8).

The observed group differences in the Empty–Bored connection may further reflect the impact of impaired insight on the experience of boredom and existential dissatisfaction. Among cognitively unimpaired older adults, boredom may strongly evoke a sense of emptiness, resulting in a tightly connected cluster of anhedonia-related symptoms. In contrast, cognitively impaired individuals may frequently experience boredom due to inactivity or limited engagement, without explicitly perceiving or articulating the associated sense of emptiness. This dissociation could contribute to the reduced centrality of emptiness in the impaired group.

Beyond these theoretical considerations, altered network structures in cognitively impaired individuals likely reflect a more complex interplay of neuropsychological and emotional processes. Cognitive deficits are frequently accompanied by disturbances in emotional processing (26, 27). Among others, these disturbances include impairments in identifying emotions in facial expressions and expressing emotions in social interactions (26, 28). Reduced autobiographical memory performance, which is common in cognitively impairment, may further limit the subjective experience and verbalization of existential feelings such as emptiness (29). Together, impairments in emotional perception, memory, emotional experience, and emotion regulation may contribute to the lower centrality of certain affective symptoms within the network (30). At the same time, neuropsychological changes may shift the depressive symptom profile toward more externally observable manifestations, such as reduced energy or psychomotor slowing. However, the precise relationship between emotional and cognitive dysfunction remains insufficiently understood (31). Future studies incorporating detailed neuropsychological assessments and functional markers may help elucidate the mechanisms underlying symptom centrality shifts.

In summary, cognitive impairment appears to shift the pattern of depressive symptoms toward more externally observable or situation-specific symptoms, such as apathy, helplessness, and restlessness, and away from internally perceived feelings of despair. This shift is likely driven by diminished insight and affective flattening.

These findings have important clinical implications. Standard depression screening instruments may underestimate depressive symptoms in cognitively impaired older adults, who are prone to under-report sadness or worthlessness. Although the GDS-15 remains a useful tool, clinicians should interpret item responses in light of cognitive deficits. Given that feelings of worthlessness emerged as a central symptom regardless of cognitive status, careful exploration of self-esteem is recommended. Conversely, reduced reporting of emptiness underscores the importance of attending to observable signs such as apathy, withdrawal, irritability, and helplessness, as well as obtaining additional information from caregivers. Screening tools and interventions that emphasize these core symptoms, in combination with structured activity programs and established antidepressant or psychotherapeutic treatments, may improve detection and management in this population (6, 32, 33).

Recognizing symptom centrality within specific subgroups may also inform intervention development. Among cognitively unimpaired older adults, where emptiness and worthlessness are central, interventions aimed at enhancing meaning, purpose, and self-esteem (e.g., behavioral activation, cognitive therapy that focuses on negative self-schemas) may exert broad effects on the network of symptoms (34). In contrast, for individuals with cognitive impairment who primarily experience helplessness or low confidence, interventions focusing on autonomy and self-efficacy may be more appropriate (35, 36). Targeting central symptoms may induce cascading improvements across connected symptoms, an assumption that warrants further empirical investigation. Nevertheless, the practical challenges of geriatric treatment and research, including time constraints in inpatient settings and limited access to outpatient psychotherapy for multimorbid, care-dependent patients, must be considered.

While this study offers valuable new insights, it is not free of limitations. First, the study sample consisted exclusively of hospitalized geriatric patients, limiting generalizability to community-dwelling or institutionalized populations. Second, the cross-sectional design precludes causal inference and limits interpretation to descriptive network patterns; longitudinal studies are needed to examine symptom dynamics over time. Third, cognitive impairment was operationalized using a broad cognitive grouping approach without differentiating between mild cognitive impairment and dementia subtypes or stages. Exclusion of individuals with a MMSE score of 16 points or less, due to validity concerns, may have introduced selection bias and restricts generalizability to those with more severe cognitive deficits. Fourth, reliance on self-reported GDS-15 data may be problematic in cognitively impaired individuals or those with subclinical delirium, despite exclusion of overt delirium. Incorporating caregiver or clinician ratings and additional geriatric symptoms relevant to geriatric depression (e.g., anxiety, irritability, sleep disturbances, pain) may yield a more comprehensive understanding of depressive symptom networks. Fifth, potentially important confounders such as educational level or medication use were not available and may have influenced both cognitive function and depressive symptoms. Finally, the monocentric design and relative demographic and cultural homogeneity of our sample may further limit generalizability.

In conclusion, older adults with cognitive impairment exhibit a distinct depressive symptom network compared to those with unimpaired cognition. These differences have important implications for screening, assessment, and treatment. Clinicians and public health professionals should adapt diagnostic and therapeutic strategies to account for the altered and often subtle presentation of depressive symptoms in cognitively impaired older adults, thereby supporting more personalized and effective approaches to geriatric mental health care.

## Data Availability

The raw data supporting the conclusions of this article will be made available by the authors, without undue reservation.
