# Supplementary material for: Depressive symptoms in hospitalized geriatric patients with and without cognitive impairment: a cross-sectional network analysis approach
Source: Front Med (Lausanne). 2026 Mar 16;13:1689283. doi: 10.3389/fmed.2026.1689283 (PMC13033779; doi:10.3389/fmed.2026.1689283)
Supplement: Supplementary file 1 [file Data_Sheet_1.pdf]

Supplementary Material

1 Supplementary Table

Supplementary Table 1. Node strength of the entire cohort.

Node	Label	Strength
G_1i	Unsatisfied	5.083
G_2	Dropped interests	2.937
G_3	Empty	5.963
G_4	Bored	2.608
G_5i	Bad spirits	4.636
G_6	Afraid	2.264
G_7i	Unhappy	4.083
G_8	Helpless	4.275
G_9	Stay home	0.606
G_10	Memory problems	1.830
G_11i	Awful	4.264
G_12	Worthless	6.262
G_13i	Lack of energy	2.610
G_14	Hopeless	4.829
G_15	Others better off	3.413

The nodes display the items of the GDS-15. GDS: Geriatric Depression Scale.

Supplementary Table 2. Sensitivity analysis of node strength after multiple imputation.

Node	Label	Strength (complete case)	Strength (multiple imputation)	Difference
G_1i	Unsatisfied	5.083	5.152	0.069
G_2	Dropped interests	2.937	3.054	0.117
G_3	Empty	5.963	6.003	0.040
G_4	Bored	2.608	2.577	-0.031
G_5i	Bad spirits	4.636	4.677	0.041
G_6	Afraid	2.264	2.303	0.040
G_7i	Unhappy	4.083	4.239	0.156
G_8	Helpless	4.275	4.235	-0.039
G_9	Stay home	0.606	0.521	-0.085
G_10	Memory problems	1.830	1.795	-0.035
G_11i	Awful	4.264	4.369	0.105
G_12	Worthless	6.262	6.248	-0.014
G_13i	Lack of energy	2.610	2.579	-0.030
G_14	Hopeless	4.829	4.662	-0.167
G_15	Others better off	3.413	3.478	0.065

The nodes display the items of the GDS-15. GDS: Geriatric Depression Scale. Sensitivity analysis using multiple imputation were performed to examine whether listwise deletion of missing GDS-15 items influenced the estimated network structure. Missing binary GDS items were imputed using logistic regression models, and networks were estimated separately in each of five imputed datasets. Node strength values were averaged across imputations and compared with those obtained from the complete-case analysis. Strength centrality estimates derived from the imputed datasets were highly similar to those obtained from the complete-case sample. Absolute differences in node strength were small across all items, ranging from -0.17 to 0.16. Importantly, the rank order of node centrality remained unchanged. *Worthlessness* (G_12) and *emptiness* (G_3) consistently emerged as the most central symptoms in both the complete-case and imputed datasets. Accordingly, these findings indicate that the primary network results were not materially affected by the handling of missing GDS-15 data, supporting the robustness of the reported network structure and centrality patterns.

Supplementary Table 3. Sensitivity analysis of node strength across different EBIC tuning parameters.

Node	Label	γ = 0.25	γ = 0.50	γ = 0.75
G_1i	Unsatisfied	5.136	5.083	5.083
G_2	Dropped interests	3.311	2.937	2.937
G_3	Empty	5.994	5.963	5.963
G_4	Bored	2.645	2.608	2.608
G_5i	Bad spirits	4.691	4.636	4.636
G_6	Afraid	2.304	2.264	2.264
G_7i	Unhappy	4.110	4.083	4.083
G_8	Helpless	4.332	4.275	4.275
G_9	Stay home	0.650	0.606	0.606
G_10	Memory problems	1.881	1.830	1.830
G_11i	Awful	4.589	4.264	4.264
G_12	Worthless	6.287	6.262	6.262
G_13i	Lack of energy	2.711	2.610	2.610
G_14	Hopeless	4.878	4.829	4.829
G_15	Others better off	3.542	3.413	3.413

The nodes display the items of the GDS-15. GDS: Geriatric Depression Scale. Sensitivity analysis using Ising models with a range of EBIC tuning parameters (γ = 0.25, 0.50, and 0.75) was performed to examine the influence of network regularization on centrality estimates. Across all specifications, node strength values showed only minor numerical variation, and the relative ordering of nodes remained fully preserved. In particular, *worthlessness* (G_12) and *emptiness* (G_3) consistently emerged as the most central symptoms. Overall, the findings demonstrate that the main results were robust across a broad range of regularization parameters and were not driven by oversparsification.

Supplementary Table 4. Sensitivity analysis of node strength using an alternative mixed graphical model.

Node	Label	Strength (IsingFit)	Strength (mgm)	Difference
G_1i	Unsatisfied	5.083	2.568	-2.515
G_2	Dropped interests	2.937	1.656	-1.282
G_3	Empty	5.963	2.965	-2.998
G_4	Bored	2.608	1.322	-1.286
G_5i	Bad spirits	4.636	2.346	-2.290
G_6	Afraid	2.264	1.152	-1.112
G_7i	Unhappy	4.083	2.055	-2.028
G_8	Helpless	4.275	2.166	-2.109
G_9	Stay home	0.606	0.325	-0.281
G_10	Memory problems	1.830	0.941	-0.889
G_11i	Awful	4.264	2.294	-1.969
G_12	Worthless	6.262	3.143	-3.119
G_13i	Lack of energy	2.610	1.356	-1.254
G_14	Hopeless	4.829	2.439	-2.390
G_15	Others better off	3.413	1.739	-1.675

The nodes display the items of the GDS-15. GDS: Geriatric Depression Scale; mgm: mixed graphical model. Sensitivity analysis using mixed graphical models (mgm) as an alternative approach to IsingFit models for the network analysis of binary data was performed. Compared with the primary IsingFit model, mgm yielded systematically lower absolute node strength values, reflecting differences in model specification and scaling. However, the relative ordering of node strength was almost identical across methods, as indicated by a near-perfect Spearman rank correlation ($\rho = 0.996$). Across both approaches, *worthlessness* (G_12) and *emptiness* (G_3) consistently emerged as the most central symptoms. These results demonstrate that the identification of central depressive symptoms was robust to the choice of network estimation method.

Supplementary Table 5. Stability of key edge weights across different EBIC tuning parameters.

**EBIC γ**	**Edge label**	**Unimpaired Cognition**	**Impaired Cogntion**
0.25	Unsatisfied – Unhappy	1.410	0.467
0.25	Empty – Bored	1.059	0.555
0.50	Unsatisfied – Unhappy	1.410	0.467
0.50	Empty – Bored	0.919	0.555
0.75	Unsatisfied – Unhappy	1.410	0.467
0.75	Empty – Bored	0.919	0.555

Sensitivity analysis with different values of the EBIC tuning parameter γ were performed to examine the stability of the two key edges identified in the network comparison test (linking *Unsatisfied* [G_1i] and *Unhappy* [G_7i], and *Empty* [G_3] and *Bored* [G_4]) in people with unimpaired cognition and impaired cognition. Across all tuning parameters, the edge connecting *unsatisfied* and *unhappy* consistently showed a markedly stronger association in people with unimpaired cognition compared with those with impaired cognition. Similarly, the association between *empty* and *bored* was consistently stronger in people with unimpaired cognition, with only minor attenuation at higher regularization levels. Importantly, variations in γ affected the absolute magnitude of edge weights but did not alter the direction or relative size of the group differences. These findings indicate that the observed edge-level differences persist across a broad range of model specifications, supporting the robustness of the reported between-group network differences.

2 Supplementary Figures

Supplementary Figure 1. Network plot of the entire cohort.

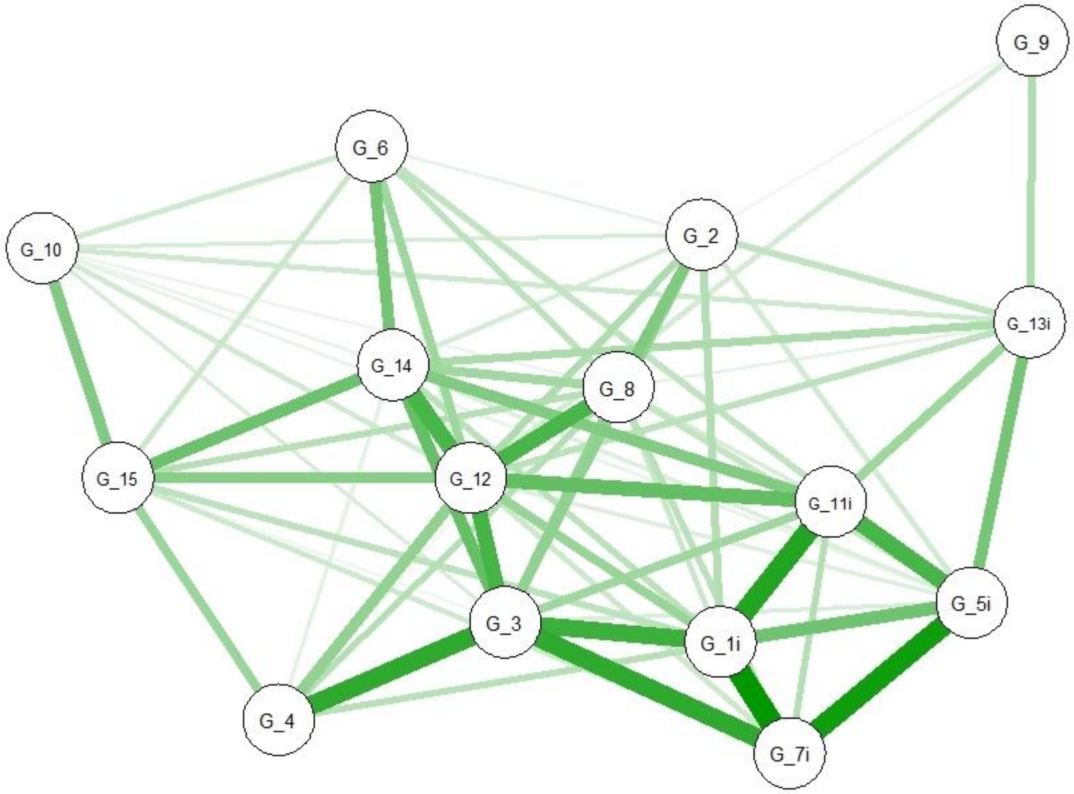

Network structure of the GDS-15 items. The nodes display the items of the GDS (G_1 – G_15). The green edges display the correlations between the nodes. The thickness of the edges indicate how strong these connections are. GDS: Geriatric Depression Scale. Item coding G_1i: Unsatisfied; G_2: Dropped interests; G_3: Empty; G_4: Bored; G_5i: Bad spirits; G_6: Afraid; G_7i: Unhappy; G_8: Helpless; G_9: Stay home; G_10: Memory problems; G_11i: Awful; G_12: Worthless; G_13i: Lack of energy; G_14: Hopeless; G_15: Others better off.

Supplementary Figure 2. Strength centrality plot of people with unimpaired cognition (UC).

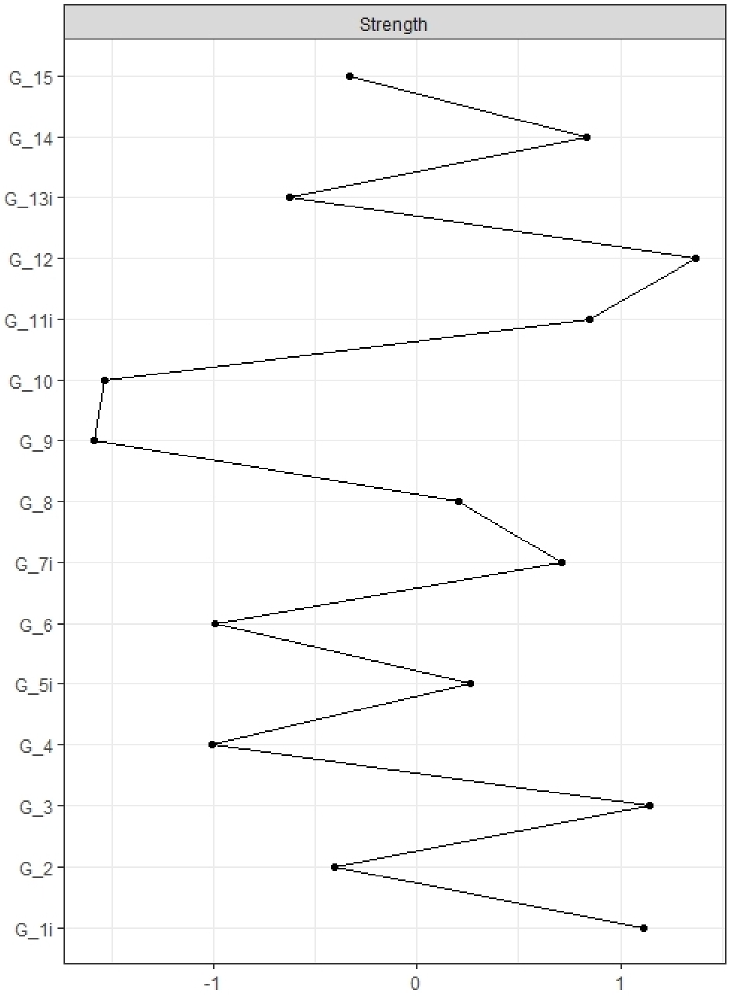

The nodes display the items of the GDS (G_1 – G_15). GDS: Geriatric Depression Scale. Item coding G_1i: Unsatisfied; G_2: Dropped interests; G_3: Empty; G_4: Bored; G_5i: Bad spirits; G_6: Afraid; G_7i: Unhappy; G_8: Helpless; G_9: Stay home; G_10: Memory problems; G_11i: Awful; G_12: Worthless; G_13i: Lack of energy; G_14: Hopeless; G_15: Others better off.

Supplementary Figure 3. Strength centrality plot of people with impaired cognition (IC).

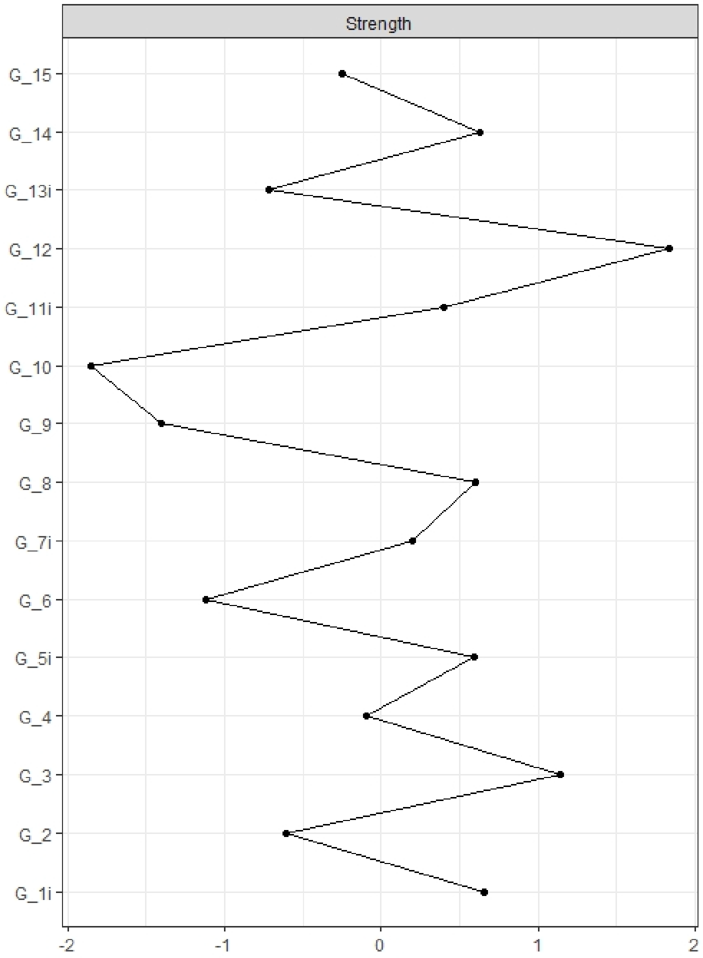

The nodes display the items of the GDS (G_1 – G_15). GDS: Geriatric Depression Scale. Item coding G_1i: Unsatisfied; G_2: Dropped interests; G_3: Empty; G_4: Bored; G_5i: Bad spirits; G_6: Afraid; G_7i: Unhappy; G_8: Helpless; G_9: Stay home; G_10: Memory problems; G_11i: Awful; G_12: Worthless; G_13i: Lack of energy; G_14: Hopeless; G_15: Others better off.

Supplementary Figure 4. Case-dropping bootstrap in people with unimpaired cognition (UC).

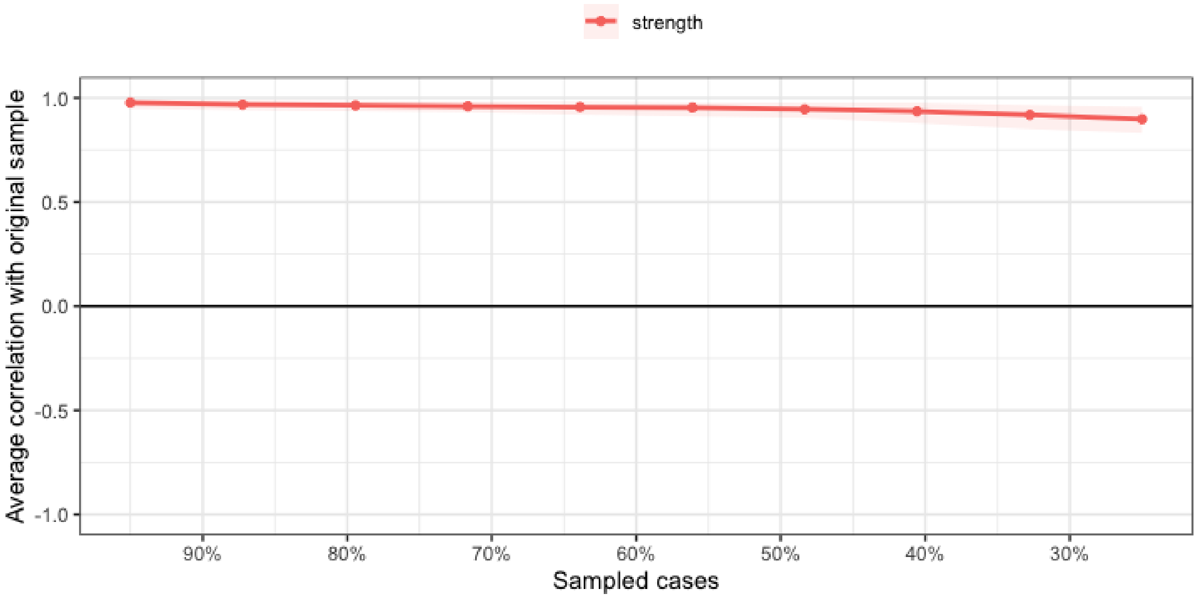

Case-dropping bootstrapped procedure of strength in people with unimpaired cognition (UC) (number of bootstraps = 1,000). The correlations of the centrality measure strength between the original sample and those from the subsamples with an increasingly higher percentage of dropout cases were calculated. The correlation stability coefficient (CS coefficient) represents the maximum proportion of cases that can be dropped to retain a correlation of at least 0.70 with the original strength in at least 95% of the samples. The 95% confidence interval of the correlation is indicated. The case-dropping bootstrap procedure showed that CS coefficient of node strength (CS(cor=0.7) = 0.75) remained high.

Supplementary Figure 5. Case-dropping bootstrap in people with impaired cognition (IC).

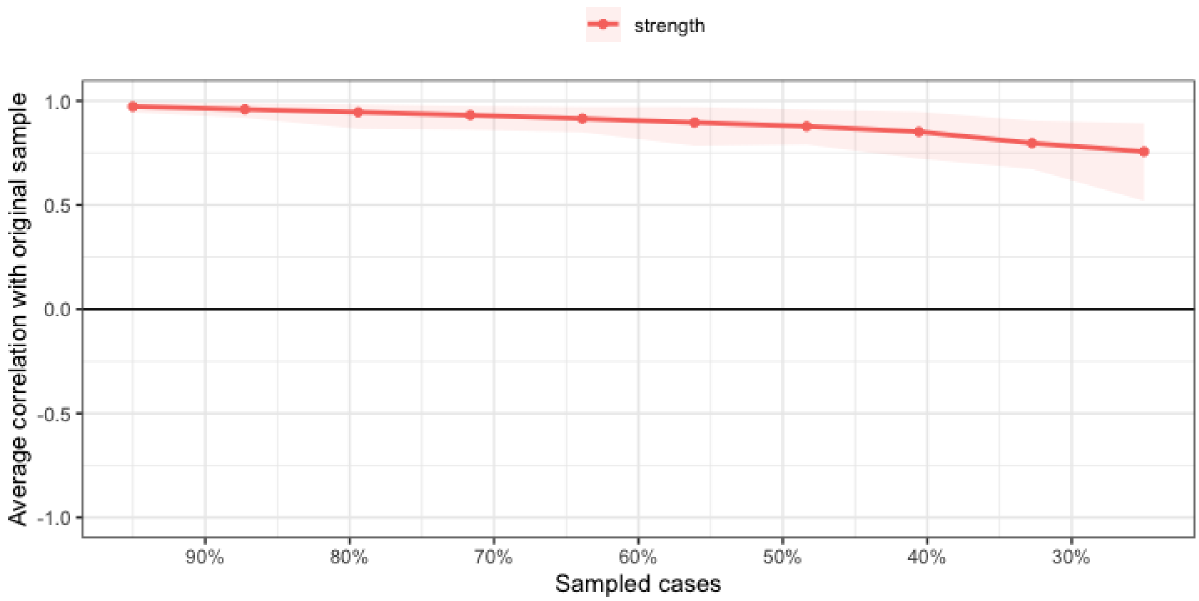

Case-dropping bootstrapped procedure of strength in people with impaired cognition (IC) (number of bootstraps = 1,000). The correlations of the centrality measure strength between the original sample and those from the subsamples with an increasingly higher percentage of dropout cases were calculated. The correlation stability coefficient (CS coefficient) represents the maximum proportion of cases that can be dropped to retain a correlation of at least 0.70 with the original strength in at least 95% of the samples. The 95% confidence interval of the correlation is indicated. The case-dropping bootstrap procedure showed that CS coefficient of node strength (CS(cor=0.7) = 0.59) remained high.
